# Serosurvey of anti-*Leishmania* (*Leishmania*) *infantum* antibodies in hunting dogs and hunters in Brazil

**DOI:** 10.14202/vetworld.2021.2735-2738

**Published:** 2021-10-24

**Authors:** Louise Bach Kmetiuk, Monique Paiva de Campos, Renato van Wilpe Bach, Ana Pérola Drulla Brandão, Ivan Roque de Barros-Filho, Leandro Cavalcante Lipinski, Giovani Marino Fávero, Andrea Pires dos Santos, Fabiano Borges Fiqueiredo, Alexander Welker Biondo

**Affiliations:** 1 Laboratory of Cellular Biology, Carlos Chagas Institute, Oswaldo Cruz Foundation, Curitiba, Paraná, Brazil; 2 Department of Medicine, State University of Ponta Grossa, Ponta Grossa, Paraná, Brazil; 3 Department of Preventive Medicine, School of Medicine, University of São Paulo, São Paulo, Brazil; 4 Department of Veterinary Medicine, Federal University of Paraná State, Curitiba, Paraná, Brazil; 5 Department of General Biology, State University of Ponta Grossa, Ponta Grossa, Paraná, Brazil; 6 Department of Comparative Pathobiology, School of Veterinary Medicine, Purdue University, Harrison Street, 725, West Lafayette, Indiana, 47907-2027, USA.

**Keywords:** Brazilian biomes, canine visceral leishmaniasis, hunting activities, *Leishmania* (*Leishmania*) *infantum*, rural dogs

## Abstract

**Background and Aim:**

Although wild boar hunting activities and the hunting dog trade in the Atlantic Forest and Cerrado biomes of Brazil overlap both with endemic and with non-endemic areas for visceral leishmaniasis, no study to date has focused on *Leishmania* spp. exposure among hunting dogs and hunters. The aim of the present study was to assess the presence of *Leishmania* spp. antibodies in hunting dogs and hunters in different anthropized areas of two Brazilian biomes.

**Materials and Methods:**

Blood samples were collected from 170 hunting dogs and 46 hunters between October 2016 and May 2018. The presence of antibodies against *Leishmania* spp. in hunting dogs was screened through a dual-path platform immunochromatographic test (DPP rapid test; Bio-Manguinhos/Fundação Oswaldo Cruz, Rio de Janeiro, Brazil) and in hunters through an rK39-based rapid immunochromatographic test. Both tests were used in accordance with Brazilian Ministry of Health recommendations.

**Results:**

Overall, although antibodies were detected through the immunochromatographic test in 3/170 (0.02%) of these female asymptomatic hunting dogs, all living in anthropized areas of the Atlantic Forest biome in South Brazil, no sample was confirmed through the enzyme-linked immunosorbent assay. All the hunters were non-reactive in the rapid immunochromatographic test.

**Conclusion:**

Our study on three suspicious hunting dogs has suggested that *Leishmania* (*Leishmania*) *infantum* may circulate both in endemic and non-endemic areas in Brazil. In addition, a high rate of hunting dog replacement due to death and trade may have led to less chance of infection and transmission between animals and between animals and humans, which would corroborate the outcomes reported here. Further studies should be conducted to fully establish whether hunting dogs and hunters may be used as sentinels in other areas endemic for *Leishmania* spp.

## Introduction

Although visceral leishmaniasis (VL) is a worldwide zoonosis, it is caused in the Americas only by the protozoon *Leishmania* (*Leishmania*) *infantum* and transmitted only by sandflies [1]. In Brazil, VL occurrences were initially reported as a rural disease, but later on, the disease spread to periurban and urban areas and increased in prevalence [1,2]. Dogs, which are considered to be the main domestic *Leishmania* reservoirs, have played an important role in its transmission in anthropized areas, which pose a high risk of infection among humans [2,3]. Not surprisingly, urban cases of VL have been associated with proximity of households to periurban wooded areas and to dogs with outdoor habits, which may connect the sylvatic to the domestic cycle [3].

In Brazil, wild boars (*Sus scrofa*) have been classified as invasive species. These animals originated from Eurasian wild boars and their hybrids, and hunting is officially permitted nationwide (Normative Instruction 03/2013) as a strategy for population control and eradication by hunters who have registered with the Brazilian Institute for the Environment and Renewable Natural Resources (IBAMA) [4]. Although the use of hunting dogs is the most popular method for wild boar tracking and active hunting nationwide in Brazil, wild boar trapping has been seen to be the alternative to hunting that fits best with animal welfare concerns [5,6]. The risks to hunting dog welfare associated with their use may include overexposure to vectors and zoonotic diseases [5], such as *Leishmania* spp.

Due to the highly adaptive capacity of wild boars, they have invaded all six Brazilian biomes [6]. Although wild boar hunting activities and the hunting dog trade in the Atlantic Forest and Cerrado biomes overlap both with endemic and with non-endemic areas for VL, no study to date has focused on *Leishmania* spp. exposure among hunting dogs and hunters. Accordingly, the aim of the present study was to assess the presence of *Leishmania* spp. antibodies in hunting dogs and hunters in different anthropized areas of two Brazilian biomes.

## Materials and Methods

### Ethical approval

This study was approved by the Ethics Committee for Animal Use (protocol number 059/2017) of the Federal University of Paraná, was officially included as part of the annual activities of the Municipal Health Department of Ponta Grossa, and was approved by National Human Ethics Research Committee (number 97639017.7.0000.0102).

### Study period and location

**Figure-1:**
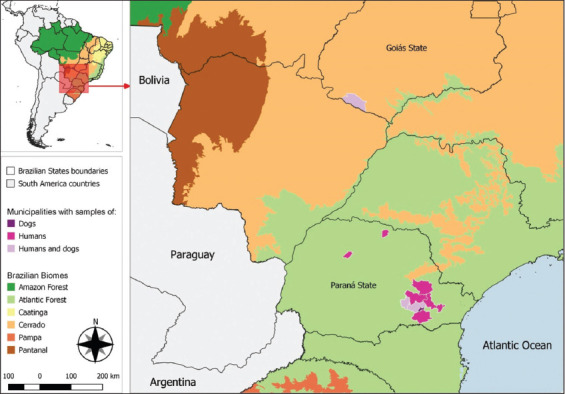
Sampling locations of hunting dogs and hunters from South and Central-West Brazil (Source: https://www.ibge.gov.br/geociencias/downloads-geociencias.html).

The study was conducted from October 2016 and May 2018. The study was conducted in anthropized areas in the Atlantic Forest biome of South Brazil, at the Paraná State, and in Cerrado biome of Central-West Brazil, at the Goiás State (Figure-1).

### Sampling

Blood samples were collected from 170 hunting dogs by means of jugular puncture and 46 hunters by means of cephalic puncture. The presence of antibodies against *Leishmania* spp. in hunting dogs was screened through a dual-path platform immunochromatographic test (DPP rapid test; Bio-Manguinhos/Fundação Oswaldo Cruz, Rio de Janeiro, Brazil), which was performed in accordance with the Brazilian Ministry of Health protocol. The results were confirmed by means of the enzyme-linked immunosorbent assay for canine VL (CVL) (EIE-LVC; Bio-Manguinhos/Fundação Oswaldo Cruz) and were interpreted as positive or negative [7]. The presence of antibodies against *Leishmania* spp. in hunters was tested through an rK39-based rapid immunochromatographic test (OnSite™ Leishmania Ab Rapid Test; CTK Biotech Inc., USA), which was performed in accordance with the Brazilian Ministry of Health protocol [8].

## Results

Overall, although antibodies were detected through the immunochromatographic test in 3/170 (0.02%) of these female asymptomatic hunting dogs, all living in anthropized areas of the Atlantic Forest biome in South Brazil, no sample was confirmed through the enzyme-linked immunosorbent assay. The three dogs with suspicious findings were born and raised in a non-endemic area of South Brazil, but two of them had been taken to areas that are endemic for CVL in Central-West Brazil, for hunting activities [9]. Unfortunately, two of these dogs with suspected CVL subsequently died during hunting and the other one was traded in Central-West Brazil, according to the two owners of these dogs, who lived no more than 20 miles apart. All the hunters were non-reactive in the rapid immunochromatographic test.

## Discussion

Even though this study only found suspicious cases, this is the first evidence of *Leishmania* spp. exposure and spreading among hunting dogs between non-endemic and endemic areas in Brazil. Although the DP^P^® CVL (Bio-Manguinhos/Fundação Oswaldo Cruz, Rio de Janeiro, Brazil) test is considered to be a screening test, its performance has shown 75.0% sensitivity and 72.9% specificity even among asymptomatic animals like the clinically healthy hunting dogs described here [10].

If these cases are considered as confirmed, the seroprevalence of *Leishmania* spp. in these hunting dogs was lower than what was found among dogs living in rural settlements in Northeast Brazil: 118/306 (38.6%) of those dogs were seropositive according to enzyme-linked immunosorbent assay [11]. However, the prevalence in our study was higher among rural dogs in a non-endemic area of South Brazil (none/689) [12]. The seroprevalence of *Leishmania* spp. among the rural dogs was higher than among urban dogs in endemic areas of Northeast Brazil, and positivity was associated with a lack of basic sanitation and garbage collection, proximity of households to forests, presence of farm animals in peridomestic areas, and accumulation of organic matter [11].

In the present study, the two anthropized areas had similar characteristics. These areas may have provided favorable conditions for sandfly species and spreading of *Leishmania* spp. One of the three suspicious hunting dogs described here had only started hunting practices around the non-endemic area where it was living. Hence, we can speculate that overlapping transit of infected hunting dogs between endemic and non-endemic areas may have predisposed them to spreading of *Leishmania* spp. in periurban scenarios. On the other hand, the high rate of hunting dog replacement due to death and trade may have led to less chance of infection and transmission between animals and between animals and humans, which would corroborate the outcomes reported here.

In addition, the hunting dogs sampled here showed moderate well-being. They were mostly in distress and kept outdoors on individual tethers and in individual kennels, with exposure to unhygienic conditions and harsh weather. Not surprisingly, a recent review on hunting dog welfare among dogs used in feral pig hunting activities in Australia identified risks such as trauma, wounds, and death during stalking; use of cruel training methods such as electronic shock collars; stress during transportation; and unsatisfactory quality of life during resting time and retirement [6]. Thus, due to such unsanitary and cruel conditions before, during, and after wild boar and feral pig hunting, dog use has been highly controversial in Brazil and other countries.

In the present study, none of the 46 hunters were seropositive for *Leishmania* spp. In a clinical study on the rK39-based rapid immunochromatographic test in Brazil, sensitivity of 91.2% and specificity of 94.5% were observed among 113 VL-positive human samples and 73 negative controls that were tested, which were lower rates than had previously been reported in India [13]. Thus, it may not be possible to extrapolate sensitivity and specificity rates to low prevalence scenarios or epidemiological surveys such as the present study. Thus, local validation is required.

Even though the present study only showed three suspicious (non-confirmed) hunting dogs, the negative results from the other 167 hunting dogs and 46 hunters should be considered important findings, particularly with regard to areas that are endemic for leishmaniasis. As previously established, Brazilian sandflies have mostly been found in periurban and transitional areas between preserved forests and urban settings [1]. As hunting dogs and hunters may routinely cross and stay within such areas during wild boar stalking and be highly exposed to *Leishmania* spp. vectors, the present findings indicate that vector and/or pathogen circulation in such areas are at low levels. Further studies should be conducted to fully establish whether hunting dogs and hunters may be used as sentinels in other areas that are endemic for *Leishmania* worldwide.

## Conclusion

Our study on three suspicious hunting dogs has suggested that *L*. (*L*.) *infantum* may circulate both in endemic and non-endemic areas in Brazil. In addition, a high rate of hunting dog replacement due to death and trade may have led to less chance of infection and transmission between animals and between animals and humans, which would corroborate the outcomes reported here. Further studies should be conducted to fully establish whether hunting dogs and hunters may be used as sentinels in other areas endemic for *Leishmania* spp.

## Authors’ Contributions

LBK, APS, FBF, and AWB: Conceptualization. LBK, MPC, IRB, RVWB, APDB, LCL, GMF, FBF, and AWB: Data collection and data analysis. LBK, MPC, FBF, and AWB: Writing original draft. All authors read and approved the final manuscript.
